# Monoamino oxidase alleles correlate with the presence of essential hypertension among hypogonadic patients

**DOI:** 10.1002/mgg3.1040

**Published:** 2019-11-19

**Authors:** José Luis Royo, Daniel Castellano‐Castillo, Maximiliano Ruiz‐Galdon, María Molina‐Vega, Fernando Cardona, Francisco J. Tinahones, José C. Fernández‐García, Armando Reyes‐Engel

**Affiliations:** ^1^ Department of Surgery, Biochemistry and Immunology School of Medicine University of Malaga Málaga Spain; ^2^ Unidad de Gestión Clínica de Endocrinología y Nutrición del Hospital Virgen de la Victoria Instituto de Investigación Biomédica de Málaga (IBIMA) Universidad de Málaga Málaga Spain; ^3^ Centro de Investigación Biomédica en Red de Fisiopatología de la Obesidad y la Nutrición, CIBERobn Málaga Spain

**Keywords:** hypertension, hypogonadism, *MAOA*, *MAOB*

## Abstract

**Background:**

Monoamine oxidase (MAO) activity has been traditionally implicated in blood pressure through its effects on biogenic amine levels such as catecholamines, serotonin, and dopamine. Nowadays, this role is considered relegated to side‐effects such as orthostatic hypotension and/or hypertensive crisis derived from MAO‐inhibitory treatments in patients with psychiatric disease.

**Methods:**

In the present work we have found an association between a polymorphic variant of *MAOB* gene and arterial hypertension in obese hypogonadic patients. The study cases comprised a series of 219 nondiabetic males with a body mass index ≥30 kg/m^2^ and aged <45 years. Hypogonadism was defined as subnormal testosterone concentrations, when free testosterone values ranged <65 pg/ml.

**Results:**

*MAOB* rs3027452‐A allele carriers were significantly over‐represented among hypertensive (HT) patients (25.49%) in comparison to either the non‐HT patients (10%, OR = 3.079 CI_95_ [1.364–6.952], *p* = .005, Chi‐square test) and the control population series of nonobese nor hypogonadic males (also 10%, *p* = .003 Chi‐square test). Upon adjusted, an independent association was shown with the hypogonadic group with hypertension when compared with nonhypertensive hypogonadics (Beta = 3.653, *p* = .005). When quantitative analysis was performed, hypertensive patients harboring rs3027452‐A allele showed higher systolic blood pressure values (*p* = .038, Mann–Whitney U‐test) as well as an increased Systolic‐Diastolic range despite following HT treatment (∆mmHg 54 vs. 48 for rs3027452‐A and rs3027452‐G respectively, *p*‐value .019, Mann–Whitney U‐test). Previous studies on *MAOB* revealed that rs3027452‐A allele has been correlated to a lower activity of the enzyme, what gives a functional evidence over our observation.

**Conclusion:**

If this result could be extrapolated to other hypertensive patient groups, it would implicate a review of the markers and therapeutic targets on human hypertension.

## Graphical abstract

Male hypogonadism is a disorder in which the testes are not functional or there is a genetic inability of the hypothalamus to secrete normal amounts of GnRH. The masculine sexual characteristics are not developed and can bring a wide range of clinical manifestations. We have found an association between a polymorphic variant of *MAOB* gene and arterial hypertension in obese hypogonadic patients.

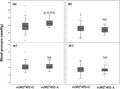

## 1 INTRODUCTION

Monoamine oxidases A and B (*MAOA* OMIM 309850 and *MAOB* OMIM 309860) genes are both located in the short arm of the X chromosome (Xp.11.4‐11.3), showing in addition a high degree of homology (Sims et al., 1992). These two genes codify for two enzymes, *MAOA* and *MAOB*, which carry out their function, mainly the degradation of bioamines such as serotonin (5‐hydroxytryptamine or 5‐HT), dopamine, and noradrenaline (Greenawalt & Schnaitman, 1970), on the external side of the mitochondrial membrane. *MAOA* predominantly catalyzes the oxidation of serotonin, whereas *MAOB* acts over 2‐phenylethylamine and benzylamine (Arai et al., 1986; Fowler & Oreland, 1981; Lenders et al., 1996). Besides, dopamine, noradrenaline, adrenaline, tryptamine, and tyramine are oxidized by both enzymes (Youdim & Bakhle, 2006). Since human lymphocytes and platelets only contain *MAOB*, this enzyme is in charge of the bioamines degradation at the circulatory level. In the brain, *MAOA* is expressed by catecholaminergic neurons (Thorpe, Westlund, Kochersperger, Abell, & Denney, 1987) whereas *MAOB* is expressed in serotonergic (Thorpe et al., 1987) and histaminergic neurons (Westlund, Denney, Rose, & Abell, 1988).

The most suitable way to determine *MAOB* activity is by analyzing thrombocyte‐MAO activity (Trbc‐MAO). It has been shown that MAO activity in the cerebral spinal fluid is associated to the serotonin activity (Oreland et al., 1981). Moreover, Trbc‐MAO activity has been described to be higher in women than in men (Harro et al., 2001). This difference in *MAOB* activity is thought to be due to a possible effect of sex steroid hormones or to an inappropriate imprinting X‐inactivation, since *MAOB* gene expression has been shown to be upregulated by a decreased DNA methylation (Good et al., 2003; Launay et al., 2009; Shih, Chen, & Ridd, 1999; Shih, Wu, & Chen, 2011). On the other hand, Trbc‐MAO activity has also been associated with several psychiatric syndromes, personality traits, and mood disorders (Shih et al., 1999).

*MAOA* and *MAOB* polymorphisms frequency have been shown to vary among different ethnic groups (Gilad, Rosenberg, Przeworski, Lancet, & Skorecki, 2002). Furthermore, several *MAOA* and *MAOB* polymorphisms have been associated to different enzymatic activities, resulting in different observed phenotypes (Jansson et al., 2005). In this sense, high‐activity genotypes have been described to increase dopamine, noradrenaline, and serotonin metabolism (Aklillu, Karlsson, Zachrisson, Ozdemir, & Agren, 2009; Andreou et al., 2014).

On the other hand, it is known the vasoactive effect that the different neuropeptides have. For instance, serotonin (which is a potent amine) has first been described as a serum vasoconstrictor molecule and subsequently has been identified as a neurotransmitter (Fraer & Kilic, 2015; Frishman et al., 1995; Sharma, Chandra, Gujrati, Shanker, & Bhargava, 1985; Struyker‐Boudier, le Noble, le Noble, Messing, & van Essen, 1990; Vanhoutte, 1991; Watts, Morrison, Davis, & Barman, 2012). Similarly, an association has been reported between dopamine metabolism and blood pressure (Hirose et al., 2013; Hunter, Boakes, Laurence, & Stern, 1970; Reder et al., 2012; Sharma et al., 1985). However, there are a very few studies about what could be the role between MAO activity and blood pressure, and to our knowledge, none of them are recent (Anselmi, Buffoni, Curradi, Del Bianco, & Sicuteri, 1976, 1974; Sicuteri, Del Bene, Anselmi, & Del Bianco, 1967; Sjoerdsma, Gillespie, & Udendriend, 1959).

Moreover, hypogonadism has been associated to hypertension. For instance, it has been described that men presenting a history of hypertension have higher prevalence of hypogonadism (Valerie Berry, 2005). It has also been reported that testosterone supplementation can lower blood pressure. This could be through an indirect action, via the decrease in adiposity, or by a direct action, via the decrease in plasma endothelin 1 (ET1, OMIM 131240), the contractile RhoA/Rho‐kinase (ROCK, OMIM 601702 and OMIM 604002) signaling pathway, the nitric oxide synthase (NOS) and an increase in the asymmetric dimethylarginine (ADMA) (Fahed, Gholmieh, & Azar, 2012). Thus, the aim of this work was to study the distribution of *MAOA* and *MAOB* polymorphisms in a group of nondiabetic obese patients with and without hypogonadism to discern the possible relationship between this genetic factor with the blood pressure in hypogonadism. This would be of clinical interest since could give clues to a better treatment of hypogonadism and its related complications as the hypertension.

## 2 MATERIALS AND METHODS

### 2.1 Study population

The study cases comprised a series of 219 nondiabetic obese males with a body mass index (BMI) ≥ 30 kg/m^2^ and aged <45 years, consecutively referred from six primary care centers from Malaga (Spain) recruited between 2013 to June 2015. Hypogonadism was defined as subnormal testosterone concentrations, defined when free testosterone values <65 pg/ml or total testosterone <3.5 ng/ml. Exclusion criteria for this study were previous diagnoses of hypogonadism, diabetes mellitus, use of antidiabetic medication, or being under any treatment known to affect the gonadal axis. Subjects with hepatic or renal impairment, established cardiovascular disease or cancer were also excluded. All subjects had a normal pubertal development and referred intact sense of smell. The control population consisted on 228 males recruited among healthy volunteers from the School of Medicine, University of Malaga. The study was reviewed and approved by the Ethics and Research Committee of Virgen de la Victoria Clinical University Hospital, Malaga (Spain), and was conducted according to the principles of the Declaration of Helsinki. The participants, all volunteers, provided signed consent after being fully informed of the study goal and its characteristics.

### 2.2 DNA extraction and genotyping

Cases provided a blood sample while healthy controls provided buccal swap that was used to extract genomic DNA using salting out method followed by ethanol precipitation. DNA quantity was determined using nanodrop prior to genotyping. Genotyping was outsourced to Genologica SL. SNP analysis was performed using the TaqMan Open Array Genotyping System from Applied Biosystems. The results obtained were processed using TaqMan Genotyper Software. The selected SNPs were chosen from the literature among those affecting the MAO activity (Gonzalez, Polvillo, Ruiz‐Galdon, Reyes‐Engel, & Royo, 2019; Xu et al., 2017; Zhang, Martin, Morris, & Li, 2009). Call ratios where >95% for every SNP.

### 2.3 Statistical analysis

Statistical analysis was performed using the SPSS statistical package (version 22 for Windows; SPSS). Bivariate regressions were used to adjust genetic Odds Ratios (OR). Multivariate results were expressed as standardized coefficient (beta). Significance was established for *p*‐values < .05. Haplotypes were estimated using THESIAS software as previously described (Tregouet & Garelle, 2007).

## 3 RESULTS

**Figure 1:**
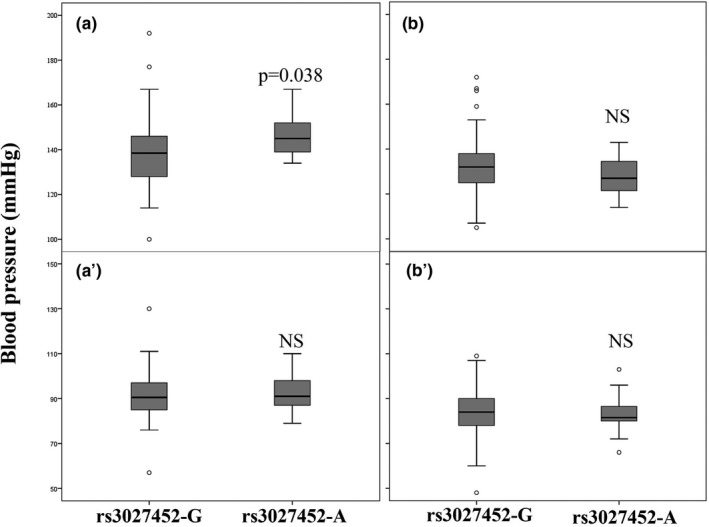
Whisker plots representing systolic (a and b) and diastolic (a' and b') of pressure according to the hypertensive status (HT: a and a', Non HT: b and b') and the rs3027452 genotype. Boxes highlight quartiles 2, 3 and 4, whereas whiskers represent the range. *p*‐value was calculated with Mann–Whitney U‐test. NS stands for not significant

In this study, we have evaluated the distribution of *MAOA* and *MAOB* polymorphisms among a hypogonadic population. No differences were observed between the genotype distribution of rs3788862, rs979605 nor rs3027452 between control population and the hypogonadic series. However, when the hypogonadic population was stratified according to the hypertensive status we found that rs3027452‐A carriers were significantly over‐represented among hypertensive (HT) patients (25.49%) in comparison to either the non‐HT patients (10%, *p* = .005, Chi‐square test) and the control population series of nonobese nor hypogonadic males (also 10%, *p* = .003 Chi‐square test; Table 1). When bivariate logistic regression was performed in an intracohort study taking into account BMI and age as covariates, the association persisted (Beta = 3.653, *p* = .005; Table 2). However, there is no difference in the genotypic distribution between the control group and the hypogonadic subjects without differentiating by their hypertensive state, nor between control normotensives and non‐HT hypogonadics. Interestingly, those HT patients harboring rs3027452‐A allele showed higher systolic blood pressure values despite that fact that they were under HT treatment (139 mmHg ± 17 for rs3027452‐G vs. 147 mmHg ± 10 for rs3027452‐A, *p*‐value = .038, Mann–Whitney U‐test; Figure 1; Table S1).

**Table 1:** Distribution of MAOA and MAOB polymorphisms in the study population

Gene	SNP	Genotype	CONTROLS	HYPOGONADICS		Controls versus hypogonadics		HT versus non HT		HT versus controls	
				Non‐HT	HT	OR [CI_95_]	*p*‐value	OR [CI_95_]	*p*‐value	OR [CI_95_]	*p*‐value
*MAOA*	rs3788862	G	166	115	34	Ref	—	Ref	—	Ref	—
		A	52	46	17	1.350 [0.879–2.072]	.169	1.250 [0.636–2.455]	.517	1.596 [0.825–3.089]	.163
	rs979605	G	122	110	35	Ref	—	Ref	—	Ref	—
		A	51	49	16	1.072 [0.692–1.663]	.755	1.026 [0.520–2.027]	.941	1.066 [0.667–1.704]	.791
*MAOB*	rs3027452	G	200	144	38	Ref	—	Ref	—	Ref	—
		A	22	16	13	1.449 [0.803–2.612]	.216	3.079 [1.364–6.952]	**.005**	3.110 [1.442–6.706]	**.003**

Abbreviations: HT, hypertensive; OR, odds ratios.

Bold values indicate results statistically significant.

**Table 2:** Bivariate logistic regression analysis of the role of *MAOB* SNP and to Hypertension

	Odds ratio	*p*‐value
*MAOA*		
rs979605	0.650	.487
rs3788862	1.890	.303
*MAOB*		
rs3027452	3.653	**.005**
Age	1.114	**<.001**
BMI	1.089	**<.001**

Bold values indicate results statistically significant.

The *MAOA* polymorphisms rs3788862 and rs979605 were in linkage disequilibrium and not associated with hypertension in hypogonadic patients. However, *MAOA* and *MAOB* polymorphism were not found in partial linkage and therefore were analyzed in combination (Table 3). The *MAOA‐MAOB* joint analysis showed that the rs3027452‐A allele had an increased Odds Ratio regardless the *MAOA* haplotype, although only with the G‐G combination was found to be statistically significant after adjusted by age and BMI. This might be a result of statistical power, since the G‐G haplotype is more prevalent than the A‐A. Thus, taking these data altogether we propose that the effect over hypertension shall be attributed to rs3027452 on its own.

**Table 3:** Genotype combinations associated to HT adjusted by age and BMI

***MAOA***		*MAOB*	Non HT (%)	HT (%)	Odds Ratio	*p*‐value
rs3788862	rs979605	rs3027452				
G‐G		G	91 (0.57)	22 (0.43)	Ref	—
A‐A		G	38 (0.24)	11 (0.22)	1.15	.753
G‐G		A	14 (0.09)	10 (0.20)	3.60	**.015**
G‐A		G	10 (0.06)	2 (0.04)	0.74	.724
A‐G		G	5 (0.03)	3 (0.22)	2.15	.368
A‐A		A	2 (0.01)	3 (0.06)	5.42	.085
Age					1.11	**<.001**
BMI					1.09	**<.001**

Abbreviations: BMI, body mass index; HT, hypertensive.

Bold values indicate results statistically significant.

## 4 DISCUSSION

In this study we report a strong association of the polymorphism rs3027452 of the *MAOB* with essential hypertension in hypogonadic patients. The effects of the activity of *MAOA* and *MAOB* on blood pressure are controversial and dual. Thus, it has been related to variations in biogenic amines levels, and in most cases induced by pharmacological agents such as serotonin reuptake inhibitors, producing, serotonergic crisis (Boyer & Shannon, 2005), treatments with Levodopa, (Murphy, 2000) and combinations of neuropeptide‐enhancing drugs such as selective serotonin reuptake inhibitors (SSRIs) and MAO inhibitors (MAOIs) in which there may be seizures in both hypo and hypertensive senses (Buu, Kuchel, Manger, Hulse, & Chute, 1986; Caston, Eaton, Gheorghiu, & Ware, 2002; Cockhill & Remick, 1987; Lefebvre, Noblet, Moore, & Wolf, 1995).

The possible relationship between neuropeptides, their receptors, and blood pressure is known (Chugh, Pokkunuri, & Asghar, 2013; Gao et al., 2014; Hirose et al., 2013; Liao et al., 2002; Murphy, 2000; Reder et al., 2012; Sicuteri et al., 1967; Svetkey et al., 1996; Watts et al., 2012; Watts et al., 2012). For instance, it has been described that MAO activity can cause blood pressure changes causing hypertensive crisis after tyramine diet intake in depressed patients treated with MAOIs (Flockhart, 2012). There are many reports of hypertensive effects or orthostatic hypotension (Golwyn & Sevlie, 1993) and even treatment of hypertension with MAOIs (Colliard, Michelet, & Tcherdakoff, 1981). However, there are few studies pointing out the association between MAO activity and essential hypertension (Anselmi et al., 1974; Cockhill & Remick, 1987; Colliard et al., 1981; Sicuteri, Buffoni, Michelacci, & Del Bene, 1969; Sicuteri et al., 1967). There are two studies describing that *MAOB* activity is diminished among patients with essential arterial hypertension compared to normotensive controls (Guicheney, Soliman, Launay, Dreux, & Meyer, 1988; Soliman, Guicheney, Launay, Meyer, & Dreux, 1987). The association between low MAO activity and hypertension is also evident in genetically induced hypertensive rats (Senjo et al., 1986). Moreover, treatment with MAO inhibitors has been shown to induce blood pressure side‐effects (Abaza & Paradis, 1968; Colliard et al., 1981; Gessa, Cuenca, & Costa, 1963), pointing out the prohypertensive effects of MAOI, or orthostatic hypotension (Cockhill & Remick, 1987; Golwyn & Sevlie, 1993; Tulen, Volkers, van den Broek, & Bruijn, 2006), which in turn has been shown to be at least in part mediated by nutritional interactions (Caston et al., 2002; Flockhart, 2012). The Renalase‐dependent degradation of catecholamines and dopamine support the evidence of the action of biogenic amines on blood pressure as well (Zbroch, Musiałowska, Koc‐Zorawska, & Malyszko, 2016). Noteworthy, polymorphisms that have been described to affect secreted Renalase levels by the kidney have been related to blood pressure (Fava et al., 2012; Wang et al., 2014).

To the beast of our knowledge, we could not find any recent study linking MAO activity with essential hypertension, neither related to genetic determinants (polymorphisms or mutations) nor to therapeutic alternatives (MAOIs). Nevertheless, a genome linkage study found that two HT *loci* within the X chromosome, the first one pinpointed between base pair 10,000,000–52,058,362 and the second between 123,825,787–168,825,787. In this sense, *MAO locus* is located between base pair 43,654,907–43,746,824 and therefore the *MAO locus* might explain the first of the two linkage results (Ciccarelli et al., 2017).

Our obese hypogonadic population is diagnosed by low levels of free testosterone (<65 pg/ml) and all of them with a BMI >30 kg/m^2^. We have not found a correlation between the studied polymorphisms and free or total testosterone levels. On the other hand, the association between sympathetic effects of obesity in the generation of HT is well known (Re, 2009). It has been shown that the main reservoir of neuropeptides are platelets and platelet *MAOB* activity can be determinant for neuropeptides levels,(Jansson et al., 2005) however, no correlation was found between the number of platelets and genotypes in this population of hypogonadic subjects with or without hypertension (data not shown).

Associations between blood pressure and the studied MAO alleles were only present in the hypogonadic HT group even though these subjects were treated with several antihypertensive drugs. Thus, systolic blood pressure was associated with the allele rs3027452‐A independently of the *MAOA* genotype. A previous work quantified MAO activity in platelets for the different *MAOB* polymorphisms (Jansson et al., 2005). They observed that the *MAOB* haplotype (C‐C‐A‐G) (rs1181252, rs2283729, rs3027452, rs1799836) was the least active in males. This haplotype is the only one that contains the *MAOB* rs3027452‐A allele, which is the polymorphism we have found to be overrepresented among our hypogonadic HT patients. Therefore, it might happen that the higher blood pressure found in these hypogonadic HT subjects could be due to the lower activity of *MAOB* associated to the A‐allele.

In conclusion we report an association between the A‐allele of the rs3027452 polymorphism for *MAOB* (which in turn can be associated to a lower *MAOB* activity) with blood pressure in obese hypogonadic patients with HT regarding the possible HT treatment. This would be of clinical interest since the assessment of genetic background in hypertension subjects could serve as starting point for personalized treatment. However, we are aware that our sample size might compromise the robustness of our findings. For this reason, further studies are necessary to replicate our results and eventually dig on the molecular mechanism by which these genetic variations can influence HT. Therefore we propose a genetic variant as a new etiological agent for HT that in turn may serve as a key target for new therapeutic approaches.

## CONFLICT OF INTEREST

The authors declare that there is no conflict of interest regarding the publication of this article.
