# Supplementary figures and images for: Monoamino oxidase alleles correlate with the presence of essential hypertension among hypogonadic patients
Source: Mol Genet Genomic Med. 2019 Nov 19;8(1):e1040. doi: 10.1002/mgg3.1040 (PMC6978270; doi:10.1002/mgg3.1040)

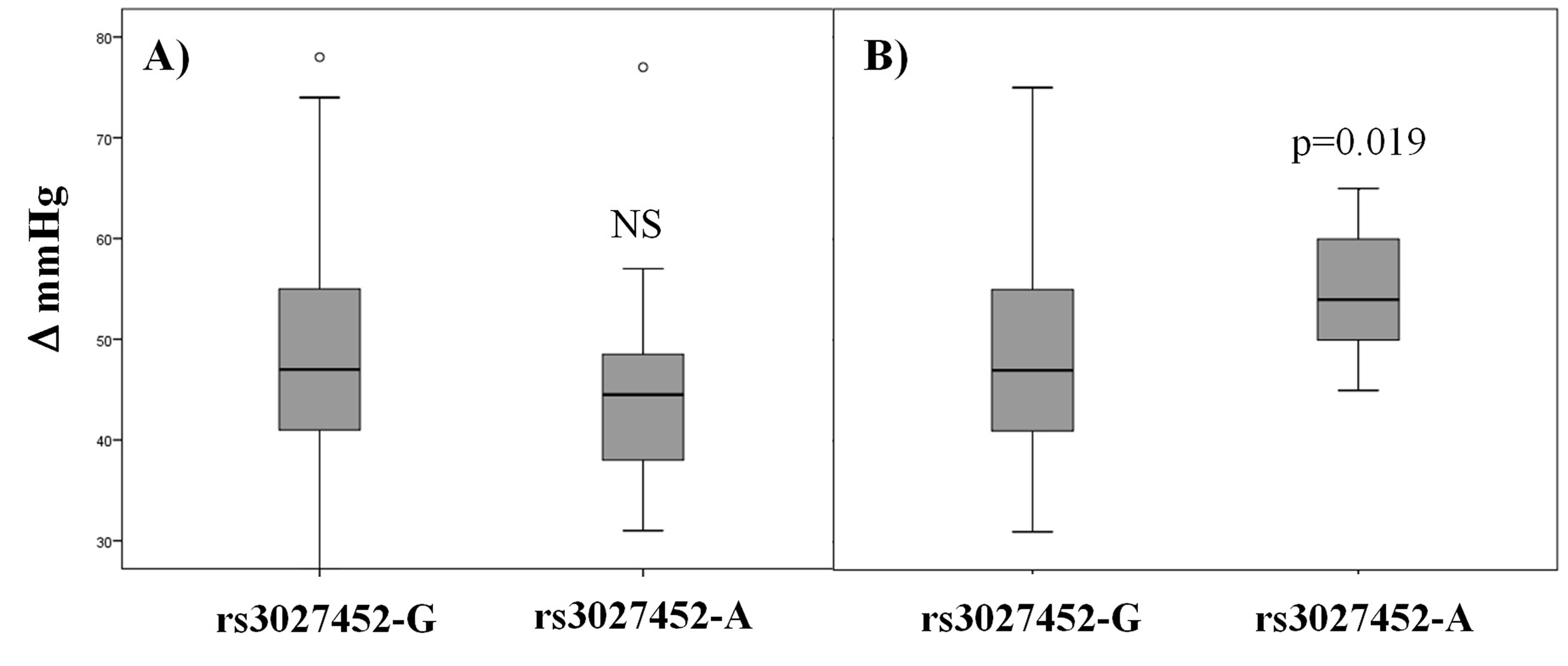

Supplement: Supplementary file 1 [file MGG3-8-e1040-s001.tif]
