# Supplementary material for: Monoamino oxidase alleles correlate with the presence of essential hypertension among hypogonadic patients
Source: Mol Genet Genomic Med. 2019 Nov 19;8(1):e1040. doi: 10.1002/mgg3.1040 (PMC6978270; doi:10.1002/mgg3.1040)
Supplement: Supplementary file 2 [file MGG3-8-e1040-s002.docx]

Table supplementary 1. Blood pressure characteristics of the two populations.

|  |  | **HT** | | | | **Non HT** | | | |
| --- | --- | --- | --- | --- | --- | --- | --- | --- | --- |
|  |  | **Systolic** | | **Diastolic** | | **Systolic** | | **Diastolic** | |
| rs3027452 | | **G** | **A** | **G** | **A** | **G** | **A** | **G** | **A** |
| Average | | 139 | 147 | 91 | 92 | 132 | 128 | 84 | 83 |
| Median | | 139 | 145 | 91 | 91 | 132 | 127 | 84 | 82 |
| CI 95% | | 133 - 144 | 141 - 153 | 87 - 95 | 88 - 97 | 130 - 134 | 123 - 132 | 83 - 86 | 78 - 83 |
| Standard Deviation | | 17 | 10 | 12 | 8 | 11 | 9 | 9 | 9 |
| Min | | 100 | 134 | 57 | 79 | 105 | 114 | 48 | 66 |
| Max | | 192 | 167 | 130 | 110 | 172 | 143 | 109 | 103 |
